# Lipid Profile Abnormalities in Type 2 Diabetes Mellitus Patients at Mbeya Zonal Referral Hospital, Tanzania: A Retrospective Study

**DOI:** 10.1155/jdr/9966933

**Published:** 2025-03-18

**Authors:** Justine Mlonga, Donath Damian

**Affiliations:** Department of Biochemistry, University of Dar es Salaam-Mbeya College of Health and Allied Sciences, Mbeya, Tanzania

**Keywords:** demographic characteristics, glycemic control, lipid profiles, Mbeya Zonal Referral Hospital, Type 2 diabetes

## Abstract

**Introduction:** Type 2 diabetes mellitus poses global health challenges due to insulin resistance and hyperglycaemia. Understanding demographic characteristics and lipid profiles among diabetic patients is crucial for effective management and risk reduction. This study analyzes demographic distribution, gender representation, and lipid profile variations among Type 2 diabetes patients at the Mbeya Zonal Referral Hospital, aiming at informing tailored interventions to improve outcomes and mitigate cardiovascular risks associated with dyslipidemia.

**Methods:** This retrospective study analyzed data from 311 Type 2 diabetes patients at the Mbeya Zonal Referral Hospital. Demographic data including age and gender were recorded, and lipid profiles (triglycerides, high-density lipoprotein, and low-density lipoprotein) were assessed using standard clinical measurements. Statistical analyses determined frequency distributions of age groups, gender proportions, and lipid profile categories. Associations between lipid profiles and demographic factors were also examined.

**Results:** The study cohort predominantly consisted of patients aged 60–69 years (35.05%), with females slightly outnumbering males (54.02% vs. 45.98%). Triglyceride levels ≥ 150 mg/dL were observed in 43% of patients, primarily in older age groups. While 72% of patients had high − density lipoprotein levels ≥ 35 mg/dL, this percentage declined with age. Low − density lipoprotein levels ≥ 130 mg/dL were prevalent in 41.77% of patients, indicating a notable proportion with elevated low-density lipoprotein cholesterol. Higher levels of high-density lipoprotein were associated with better glycemic control, as indicated by lower glycated hemoglobin levels (< 6.5%), although the relationship between high-density lipoprotein and cardiovascular outcomes remains unclear.

**Conclusion:** This study reveals significant demographic and lipid profile variations among Type 2 diabetes patients at the Mbeya Zonal Referral Hospital, influenced by aging. Tailored management strategies considering age- and gender-specific trends in lipid profiles could optimize glycemic control and reduce cardiovascular risks associated with Type 2 diabetes, thereby enhancing overall patient outcomes and quality of life.

## 1. Introduction

Type 2 diabetes mellitus (T2DM) is a chronic metabolic disorder characterized by insulin resistance and relative insulin deficiency, leading to hyperglycemia and various systemic complications. Among these complications, dyslipidemia, characterized by abnormalities in lipid metabolism, is prevalent and significantly contributes to the heightened cardiovascular risk associated with T2DM [1]. Lipid profile abnormalities, including elevated triglycerides (TGs), decreased high-density lipoprotein cholesterol (HDL-C), and increased low-density lipoprotein cholesterol (LDL-C), are commonly observed in individuals with T2DM [1]. Understanding the specific lipid profile abnormalities prevalent in T2DM patients is crucial for effective management and prevention of cardiovascular complications.

The urgency of T2DM is underscored by its high incidence, morbidity, and mortality rates. Globally, approximately 537 million adults are affected by diabetes, with projections indicating that this number will rise to 783 million by 2045 [1]. In the United States alone, about 34.2 million people have diabetes, with 90%–95% of these cases being T2DM [1]. T2DM significantly impacts morbidity, contributing to a two- to fourfold increased risk of cardiovascular disease, which is a leading cause of mortality among T2DM patients. Cardiovascular disease accounts for approximately 60%–80% of diabetes-related deaths [1, 2].

Elevated TG levels represent a hallmark of dyslipidemia in T2DM patients, often attributed to insulin resistance and impaired TG metabolism. Insulin resistance impedes the ability of insulin to suppress lipolysis in adipose tissue, leading to increased free fatty acid release into the bloodstream and subsequent TG synthesis in the liver. This dysregulated lipid metabolism contributes to elevated TG levels, which are recognized as an independent risk factor for cardiovascular disease [2].

Conversely, decreased HDL-C levels are frequently observed in T2DM patients and are associated with an increased risk of cardiovascular events. HDL-C plays a crucial role in reverse cholesterol transport, removing excess cholesterol from peripheral tissues and transporting it back to the liver for excretion. In individuals with T2DM, various factors, including insulin resistance and oxidative stress, impair HDL-C functionality, leading to reduced cholesterol efflux capacity and increased susceptibility to atherosclerosis [3].

Moreover, alterations in LDL-C levels further exacerbate cardiovascular risk in T2DM patients. While LDL-C levels may not always be elevated, T2DM patients often exhibit atherogenic dyslipidemia characterized by smaller, denser LDL particles that are more prone to oxidation and endothelial dysfunction. These modified LDL particles contribute to the development of atherosclerosis, the underlying pathology of most cardiovascular events [4].

Assessing lipid profile abnormalities in T2DM patients is essential for risk stratification and guiding therapeutic interventions aimed at reducing cardiovascular risk. Regular monitoring of lipid profiles enables clinicians to identify individuals at higher risk of cardiovascular complications and tailor treatment strategies accordingly. Lifestyle modifications, including dietary interventions and exercise, play a fundamental role in managing dyslipidemia in T2DM, complemented by pharmacological interventions such as statins and fibrates to optimize lipid levels and mitigate cardiovascular risk [1].

In this context, a retrospective study conducted at the Mbeya Zonal Referral Hospital is aimed at elucidating the specific lipid profile abnormalities prevalent in T2DM patients within the region. By analyzing lipid profiles of T2DM patients, the study seeks to provide valuable insights into the dyslipidemia patterns and associated cardiovascular risk in this population. Identifying lipid profile abnormalities in T2DM patients at the Mbeya Zonal Referral Hospital can help tailor management strategies for individuals, ultimately improving patient outcomes and reducing the risk of cardiovascular disease in this population.

## 2. Research Methodology

### 2.1. Study Design

This study employed a retrospective observational design to analyze lipid profile abnormalities in patients diagnosed with T2DM at the Mbeya Zonal Referral Hospital. Retrospective data was collected from medical records of T2DM patients who visited the hospital over a specified period from November 2022 to June 2024.

### 2.2. Study Setting

The study was conducted at the Mbeya Zonal Referral Hospital, located in Mbeya, Tanzania. This hospital serves as a tertiary healthcare facility and provides specialized services to patients from Mbeya Region and neighboring areas.

### 2.3. Study Population

The study population consisted of all T2DM patients who visited the Mbeya Zonal Referral Hospital from November 2022 to June 2024. Patients of all ages and genders with a confirmed diagnosis of T2DM were included in the study.

### 2.4. Inclusion Criteria

The inclusion criteria are patients diagnosed with T2DM confirmed by medical records at the Mbeya Zonal Referral Hospital and patients with medical records with complete information on lipid profile measurements, including total cholesterol, TGs, HDL-C, and LDL-C.

### 2.5. Exclusion Criteria

The exclusion criteria are patients with Type 1 diabetes mellitus or other forms of diabetes and patients with medical records with incomplete or missing data on lipid profile measurements, glycemic control, or medication history.

### 2.6. Estimated Study Size Calculation

Based on previous studies and hospital records, an estimated sample size of 300 T2DM patients was calculated using a power analysis to achieve a confidence level of 95% and a power of 80% for detecting significant associations between lipid profile abnormalities and cardiovascular risk. The sample size calculation considered an expected prevalence of dyslipidemia of 50% and a margin of error of 5%.

## 3. Age Group Classification Rationale

In this study, diabetic patients were categorized into specific age groups (30–39, 40–49, 50–59, 60–69, 70–79, 80–89, and 90–99 years) to capture detailed demographic trends and variations in diabetes management across different life stages. These age brackets were selected based on clinical relevance and standard epidemiological practices, allowing for precise analysis of diabetes prevalence and tailored healthcare strategies. This approach aligns with common classifications used in research and clinical guidelines to better understand and address the diverse needs of diabetic patients.

### 3.1. Data Collection

Data was collected retrospectively from medical records of T2DM patients using a standardized data collection form. The following variables will be extracted from the records: demographic information (age and gender), laboratory parameters: lipid profile (total cholesterol, TGs, HDL-C, and LDL-C), and glycemic control measures (HbA1C).

### 3.2. Data Analysis

Descriptive statistics was used to summarize the demographic and clinical characteristics of the study population. Continuous variables were presented as means with standard deviations or medians with interquartile ranges, depending on the distribution of data. Categorical variables were presented as frequencies and percentages. The prevalence of lipid profile abnormalities (elevated TGs, decreased HDL-C, and altered LDL-C) was determined.

### 3.3. Ethical Considerations

This study adhered to ethical principles outlined in the Declaration of Helsinki. Ethical approval was obtained from the University of Dar es Salaam Mbeya College of Health and Allied Sciences Review Board (Approval Number 2020-04-12994), and permission was obtained from the Mbeya Zonal Referral Hospital before data collection. Patient confidentiality was ensured by deidentifying data during analysis and reporting. Consent for publication was obtained from the relevant authorities at the Mbeya Zonal Referral Hospital.

### 3.4. Potential Confounders and Control Measures

To ensure accurate results, the study acknowledged potential confounders that could influence the findings, including comorbid conditions such as hypertension and renal disease, lifestyle factors like diet and physical activity, variations in medication adherence, and potential differences in laboratory techniques or measurement errors. To address these confounders, multivariable regression analysis was utilized. This statistical approach adjusted for variables such as age, gender, comorbid conditions, and glycemic control measures, thereby isolating the specific impact of lipid profile abnormalities on cardiovascular risk. Additionally, standardized data collection forms and trained personnel were employed to maintain consistency and minimize biases during data extraction.

### 3.5. Efforts to Address Potential Sources of Bias

To minimize potential sources of bias, a standardized data collection form was used to ensure consistency in data extraction. Data collection was performed by trained personnel to reduce variability. Additionally, the study excluded patients with incomplete or missing data to avoid biases introduced by incomplete records. Data anonymization and deidentification were employed to ensure confidentiality and mitigate selection bias.

## 4. Results and Discussion

### 4.1. Demographic Profile of Diabetic Patients at the Mbeya Zonal Referral Hospital

The data from diabetic patients attending the Mbeya Zonal Referral Hospital shows a diverse age distribution. The predominant age group is 60–69 years, with 109 patients, representing 35.05% of the total patient population. This is followed by the 50–59 years' group, which includes 84 patients (27.01%). The 70–79 years' group has 47 patients, making up 15.11% of the total. Conversely, the 30–39 years' group is the smallest, with only 11 patients (3.54%). The 80–89 years' group has 13 patients (4.18%), and the 90–99 years' group is the smallest in terms of numbers, with just one patient, representing 0.32% (Table 1).

Regarding gender distribution, the hospital's diabetic patient population consists of 143 males (45.98%) and 168 females (54.02%). This indicates a slightly higher proportion of female patients compared to male patients. Overall, the gender distribution is relatively balanced, but females are slightly more represented in this patient cohort.

### 4.2. TG Level Data for Type 2 Diabetes Patients at the Mbeya Zonal Referral Hospital

In the cohort of Type 2 diabetes patients at the Mbeya Zonal Referral Hospital, 56 male patients have TG levels of 150 mg/dL or higher, while 87 male patients have levels below 150 mg/dL. For female patients, 78 have TG levels ≥ 150 mg/dL, and 90 have levels < 150 mg/dL. Overall, the total number of patients with TG levels ≥ 150 mg/dL is 134, which constitutes 43% of the total patient population, whereas 177 patients, or 57%, have TG levels below 150 mg/dL (Table 2).

The distribution of TG levels across different age groups shows that the prevalence of elevated TG levels increases with age. In the 30–39 age group, 4 patients have TG levels ≥ 150 mg/dL and 7 have levels < 150 mg/dL. In the 40–49 age group, 21 patients have TG levels ≥ 150 mg/dL and 25 have levels < 150 mg/dL. For the 50–59 age group, 40 patients have TG levels ≥ 150 mg/dL, and 44 have levels < 150 mg/dL. In the 60–69 age group, 47 patients have TG levels ≥ 150 mg/dL, while 62 have levels < 150 mg/dL. The trend continues in the 70–79 age group with 16 patients having levels ≥ 150 mg/dL and 31 having levels < 150 mg/dL. For the 80–89 age group, 5 patients have levels ≥ 150 mg/dL and 8 have levels < 150 mg/dL. In the 90–99 age group, one patient has TG levels ≥ 150 mg/dL, and no patients have levels < 150 mg/dL (Table 2).

The total count of patients with TG levels ≥ 150 mg/dL across all age groups is 134, which represents 43% of the patient population, while 177 patients, or 57%, have TG levels < 150 mg/dL. This distribution highlights an increasing trend of elevated TG levels with age, emphasizing the need for targeted management strategies in older age groups.

### 4.3. High-Density Lipoprotein Level Data for Type 2 Diabetes Patients at the Mbeya Zonal Referral Hospital

Among the Type 2 diabetes patients at the Mbeya Zonal Referral Hospital, a total of 247 individuals have HDL-C levels at or above 35 mg/dL, representing 72% of the patient population. In contrast, 64 patients, or 28%, have HDL-C levels below 35 mg/dL. Breaking it down by gender, 105 male patients have HDL‑C levels ≥ 35 mg/dL and 38 have levels < 35 mg/dL. For female patients, 142 have HDL‑C levels ≥ 35 mg/dL, while 26 have levels < 35 mg/dL (Table 3).

The distribution of HDL-C levels across different age groups reveals that, overall, 247 patients have HDL levels ≥ 35 mg/dL and 64 patients have HDL‑C levels < 35 mg/dL. In the age group of 30–39 years, 9 patients have HDL‑C levels ≥ 35 mg/dL and 2 have levels < 35 mg/dL. For the 40–49 age group, 34 patients have HDL‑C levels ≥ 35 mg/dL, while 12 have levels < 35 mg/dL. In the 50–59 age group, 68 patients have HDL‑C levels ≥ 35 mg/dL and 16 have levels < 35 mg/dL. The trend continues with 87 patients in the 60–69 age group having HDL‑C levels ≥ 35 mg/dL and 22 having levels < 35 mg/dL. For the 70–79 age group, 38 patients have HDL‑C levels ≥ 35 mg/dL and 9 have levels < 35 mg/dL. In the 80–89 age group, 10 patients have HDL‑C levels ≥ 35 mg/dL and 3 have levels < 35 mg/dL. Finally, in the 90–99 age group, one patient has HDL‑C levels ≥ 35 mg/dL, while no patient has levels < 35 mg/dL (Table 3).

This data highlights the HDL-C level distribution among Type 2 diabetes patients, showing a higher proportion of patients with HDL-C levels below 35 mg/dL, especially as age increases.

### 4.4. HbA1C Levels With HDL-C Level Data for Type 2 Diabetes Patients at the Mbeya Zonal Referral Hospital

The data on HbA1C levels in relation to HDL-C levels among Type 2 diabetes patients at the Mbeya Zonal Referral Hospital reveals significant insights. For patients with HDL-C levels greater than 35 mg/dL, there are 79 individuals who have HbA1C levels below 6.5%, while 168 individuals have HbA1C levels at or above 6.5%. This totals to 247 patients with HDL-C levels above 35 mg/dL.

In contrast, among patients with HDL-C levels of 35 mg/dL or less, there are 21 individuals with HbA1C levels below 6.5% and 43 individuals with HbA1C levels at or above 6.5%. This totals to 64 patients with HDL-C levels of 35 mg/dL or less (Table 4).

Overall, the dataset includes 311 patients. Out of these, 100 patients have HbA1C levels below 6.5%, and 211 patients have HbA1C levels at or above 6.5%. This distribution highlights the relationship between HDL-C levels and glycemic control among patients with Type 2 diabetes, suggesting a potential association between higher HDL-C levels and better glycemic control (Table 4).

### 4.5. LDL-C Level Data for Type 2 Diabetes Patients at the Mbeya Zonal Referral Hospital

In the LDL-C level data for Type 2 diabetes patients at the Mbeya Zonal Referral Hospital, the distribution varies significantly by gender and age group. For gender-based LDL-C levels, 57 male patients have LDL-C levels less than 100 mg/dL, 36 have LDL-C levels between 100 and 129 mg/dL, and 50 have LDL-C levels of 130 mg/dL or higher. In comparison, 46 female patients have LDL‑C levels < 100 mg/dL, 42 have LDL levels between 100 and 129 mg/dL, and 80 have LDL‑C levels ≥ 130 mg/dL. This results in a total of 103 patients with LDL‑C levels < 100 mg/dL, 78 patients with LDL-C levels between 100 and 129 mg/dL, and 130 patients with LDL‑C levels ≥ 130 mg/dL, making up a total of 311 patients (Table 5).

When examining LDL-C levels by age group, the data shows the following distribution: In the 30–39 age group, 4 patients have LDL‑C levels < 100 mg/dL, 2 have LDL-C levels between 100 and 129 mg/dL, and 3 have LDL‑C levels ≥ 130 mg/dL. For the 40–49 age group, 12 patients have LDL‑C levels <100 mg/dL, 13 have levels between 100 and 129 mg/dL, and 21 have levels ≥ 130 mg/dL. In the 50–59 age group, 25 patients have LDL‑C levels < 100 mg/dL, 23 have LDL-C levels between 100 and 129 mg/dL, and 36 have levels ≥ 130 mg/dL. The 60–69 age group shows 36 patients with LDL‑C levels < 100 mg/dL, 33 with levels between 100 and 129 mg/dL, and 40 with levels ≥ 130 mg/dL. For the 70–79 age group, 21 patients have LDL‑C levels < 100 mg/dL, 5 have levels between 100 and 129 mg/dL, and 21 have levels ≥ 130 mg/dL. The 80–89 age group includes 5 patients with LDL‑C levels < 100 mg/dL, 1 with levels between 100 and 129 mg/dL, and 7 with levels ≥ 130 mg/dL. Finally, in the 90–99 age group, there are no patients with LDL‑C levels < 100 mg/dL, one patient with LDL-C levels between 100 and 129 mg/dL, and no patients with levels ≥ 130 mg/dL (Table 5). This breakdown illustrates the LDL-C cholesterol distribution among patients, highlighting both gender-specific and age-specific trends in cholesterol levels.

### 4.6. Total Cholesterol (TCHOL) Level Among Type 2 Patients at MZRH

In the TCHOL level data for Type 2 diabetes patients at MZRH, the distribution varies significantly by age group and gender. For gender-based TCHOL levels, 95 male patients have TCHOL levels less than 200 mg/dL, 30 have TCHOL between 200 and 240 mg/dL, and 18 have TCHOL level of 240 mg/dL or higher. In comparison, 92 female patients have TCHOL of less than 200 mg/dL, 46 have TCHOL levels between 200 and 240 mg/dL, and 30 have TCHOL level of 240 mg/dL or higher. This results in total of 187 patients with TCHOL less than 200 mg/dL, 76 patients with TCHOL level between 200 and 240 mg/dL, and 48 with TCHOL level of 240 mg/dL and above, making up a total of 311patients (Table 6).

When examining TCHOL levels by age group, the data shows the following distribution: In the 30–39 age group, 7 patients have TCHOL levels < 200 mg/dL, 2 have TCHOL levels between 200 and 240 mg/dL, and 2 have TCHOL levels ≥ 240 mg/dL. For the 40–49 age group, 26 patients have TCHOL levels < 200 mg/dL, 11 have TCHOL levels between 200 and 240 mg/dL, and 9 have levels ≥ 240 mg/dL. In the 50–59 age group, 49 patients have TCHOL levels < 200 mg/dL, 23 have TCHOL levels between 200 and 240 mg/dL, and 12 have levels ≥ 240 mg/dL. The 60–69 age group shows 69 patients with TCHOL levels < 200 mg/dL, 24 with levels between 200 and 240 mg/dL, and 16 with levels ≥ 240 mg/dL. For the 70–79 age group, 29 patients have TCHOL levels < 200 mg/dL, 12 have levels between 200 and 240 mg/dL, and 6 have levels ≥ 240 mg/dL. The 80–89 age group includes 6 patients with TCHOL levels < 200 mg/dL, 4 with levels between 200 and 240 mg/dL, and 3 with levels ≥ 240 mg/dL. Finally, in the 90–99 age group, there are one patient with TCHOL levels < 200 mg/dL, no patient with TCHOL levels between 200 and 240 mg/dL, and no patients with levels ≥ 240 mg/dL (Table 6). The breakdown illustrates TCHOL cholesterol distribution among patients, highlighting both gender-specific and age-specific trends in TCHOL levels.

### 4.7. Mean and Median Lipid Values by Age Group Among Type 2 Patients at MZRH

**Figure 1:**
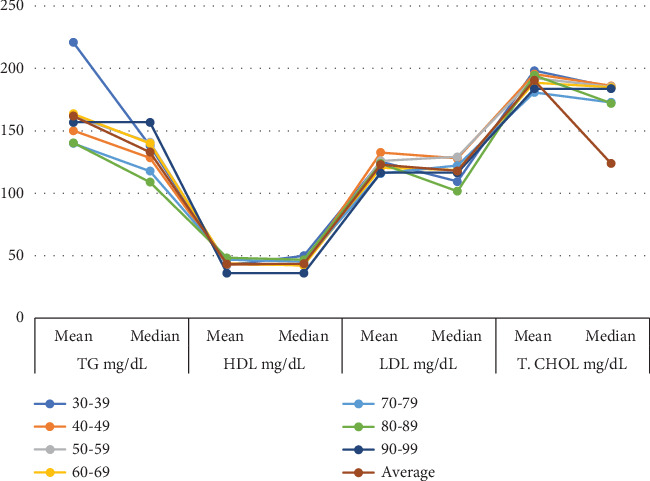
Mean and median lipid values by age group among Type 2 patients at MZRH.

The results indicate that both mean and median values for TG decrease with age, with the highest levels observed in the 30–39 age group and the lowest in the 70–79 group. HDL-C values remain relatively stable across all age groups, showing only slight variation in both mean and median values. LDL-C levels decline with age, with both mean and median values decreasing from the 30–39 age group to the 70–79 group. Similarly, TCHOL shows a slight decrease in mean and median values as individuals age, with the highest levels observed in younger age groups. In summary, TG and LDL-C levels decrease with age, while HDL-C remains stable, and TCHOL declines slightly (Figure 1).

## 5. Discussion

The demographic profile of Type 2 diabetes patients at the Mbeya Zonal Referral Hospital highlights a significant prevalence among older age groups, particularly those aged 60–69 years. This finding aligns with a broad body of epidemiological literature identifying aging as a key risk factor for Type 2 diabetes [5, 6]. Previous studies have consistently shown that the prevalence of diabetes increases with age, reinforcing the notion that older adults are more susceptible to this chronic condition. The notably low proportion of patients in the 30–39-year age bracket is consistent with global trends, indicating that Type 2 diabetes is less prevalent among younger individuals, which is corroborated by similar studies [7].

In terms of gender distribution, the slight predominance of females (54.02%) observed in this study is consistent with research that finds a higher prevalence of Type 2 diabetes among women [8]. Various factors may contribute to this gender difference, including hormonal and metabolic variations, though the degree of disparity can vary across different populations [9]. The finding aligns with literature suggesting that gender-specific factors influence diabetes prevalence, but the variation across different regions and studies highlights the need for further investigation into localized factors.

The TG level data indicate that 43% of patients have levels ≥ 150 mg/dL, which supports existing research that links elevated TG levels with aging [10]. The observation that TG levels increase with age is consistent with other studies that emphasize the progressive nature of lipid abnormalities in older adults [11]. Additionally, the higher prevalence of elevated TGs among females compared to males corroborates studies highlighting gender differences in lipid profiles [8, 9]. These differences may be attributed to variations in lipid metabolism between genders.

Regarding HDL levels, this study found that 72% of patients have HDL‑C levels ≥ 35 mg/dL, which is consistent with research indicating that higher HDL-C levels are more common among women [12]. The trend of increasing HDL-C levels with age supports findings from other studies, though the higher proportion of patients with HDL‑C levels < 35 mg/dL in older age groups highlights a discrepancy. This discrepancy underscores the need for targeted cholesterol management strategies, particularly in the elderly population [13].

The association between HDL-C levels and HbA1C levels in this study suggests that higher HDL-C levels are linked to better glycemic control, aligning with research that identifies HDL-C as a factor influencing glycemic management [12, 14]. The higher percentage of patients with HbA1C levels < 6.5% among those with HDL‑C > 35 mg/dL supports the idea that improving HDL-C levels may enhance diabetes management.

Data on LDL-C levels reveal a higher prevalence of elevated LDL-C levels among females compared to males, consistent with studies reporting gender-specific differences in lipid profiles [14]. The age-related increase in LDL-C levels observed in this study further corroborates research linking elevated LDL-C levels with advancing age [15]. This highlights the importance of monitoring and managing LDL-C levels, especially in older patients.

The TCHOL level data show that 36.7% of patients have elevated TCHOL levels (≥ 240 mg/dL), consistent with findings from other studies that report similar lipid profile abnormalities in Type 2 diabetes patients [16]. The age-related increase in TCHOL levels aligns with literature showing a correlation between aging and higher TCHOL levels [17]. This underscores the need for age-specific strategies in managing cholesterol levels in diabetic patients.

Overall, these findings provide a comprehensive view of the lipid profiles and glycemic control in Type 2 diabetes patients at the Mbeya Zonal Referral Hospital. The data emphasize the importance of tailored management approaches that consider age, gender, and individual lipid profiles to optimize diabetes care.

### 5.1. Limitations of the Study

This study has several limitations that should be considered when interpreting the findings. First, the cross-sectional nature of the study limits the ability to establish causality between age, gender, lipid profiles, and glycemic control. Longitudinal studies would be beneficial to assess the temporal relationships and causative factors more comprehensively. Second, the study sample is drawn from a single referral hospital, which may limit the generalizability of the results to other settings or populations. Variations in healthcare access, lifestyle, and genetic factors across different regions could influence the prevalence and management of Type 2 diabetes. Additionally, self-reported data and retrospective chart reviews may introduce biases or inaccuracies in the recorded lipid profiles and glycemic control measures. Finally, while this study provides valuable insights into the demographic and clinical characteristics of Type 2 diabetes patients, it does not account for all potential confounding variables, such as medication use, physical activity levels, and dietary habits, which could also impact lipid profiles and diabetes management. Future research should address these limitations to enhance the understanding of Type 2 diabetes in diverse populations.

## 6. Conclusion

The analysis of Type 2 diabetes patients at the Mbeya Zonal Referral Hospital reveals that the disease predominantly affects older adults, with a notable increase in TG and LDL-C levels in this group. Gender differences show slightly more females affected, and HDL-C levels, while generally favourable, need improvement, especially in older patients. Effective management of lipid profiles, considering age and gender, is crucial for better diabetes control and reduced cardiovascular risk.

## Figures and Tables

**Table 1:** Demographic profile of diabetic patients at the Mbeya Zonal Referral Hospital.

**Variable**	**Category**	**Frequency**	**Percentage**	
Age	30–39	11	3.54%	
	40–49	46	14.79%	
	50–59	84	27.01%	
	60–69	109	35.05%	
	70–79	47	15.11%	
	80–89	13	4.18%	
	90–99	1	0.32%	
	Total	311	100%	
Gender	Male	143	45.98%	
	Female	168	54.02%	
	Total	311	100%	

**Variable**	**Category frequency**			
Mean BMI	< 25	25–29	≥ 30	Total
	98 (31.5%)	206 (66.2%)	7 (2.3%)	311

**Table 2:** Triglyceride level data for Type 2 diabetes patients at the Mbeya Zonal Referral Hospital.

**Gender**	**Triglyceride level ≥ 150 mg/dL**	**Triglyceride level < 150 mg/dL**	
Male	56	87	
Female	78	90	
Total	134 (43%)	177 (57%)	

**Age group**	**Triglyceride level ≥ 150 mg/dL**	**Triglyceride level < 150 mg/dL**	**Mean triglyceride level (mg/dL)**
30–39	4	7	220.92
40–49	21	25	150.16
50–59	40	44	161.67
60–69	47	62	163.74
70–79	16	31	140.03
80–89	5	8	140.42
90–99	1	0	156.77
Total	134 (43%)	177 (57%)	161.96

**Table 3:** High-density lipoprotein level data for Type 2 diabetes patients at the Mbeya Zonal Referral Hospital.

**Gender**	**H** **D** **L**‑**C** **level** ≥ 35	**H** **D** **L**‑**C** **level** < 35	
Male	105	38	
Female	142	26	
Total	247 (72%)	64 (28%)	

**Age group**	**H** **D** **L**‑**C** **level** ≥ 35	**H** **D** **L**‑**C** **level** < 35	**Mean HDL-C level (mg/dL)**
30–39	9	2	42.37
40–49	34	12	42.86
50–59	68	12	43.29
60–69	87	22	43.68
70–79	38	9	46.96
80–89	10	3	48.20
90–99	1	0	35.96
Total	247	64	Mean 43.33

**Table 4:** HbA1C levels with HDL-C level data for Type 2 diabetes patients at the Mbeya Zonal Referral Hospital.

**HDL-C level**	**H** **b** **A**1**C** < 6.5	**H** **b** **A**1**C** ≥ 6.5	**Total**
> 35	79	168	247
≤ 35	21	43	64
Total	100 (32.2%)	211 (67.8%)	311

**Table 5:** LDL-C level data for Type 2 diabetes patients at the Mbeya Zonal Referral Hospital.

**Gender**	**L** **D** **L**‑**C** < 100	**L** **D** **L**‑**C** ≥ 100** and < 130**		**L** **D** **L**‑**C** ≥ 130	**Total**
Male	57	36		50	143
Female	46	42		80	168
Total	103 (33.3%)	78 (25%)		130 (41.8%)	311

**Age group**	**L** **D** **L**‑**C** < 100	**L** **D** **L**‑**C** ≥ 100** and < 130**	**L** **D** **L**‑**C** ≥ 130	**Total**	**Mean LDL-C level (mg/dL)**
30–39	4	2	3	9	125.40
40–49	12	13	21	46	132.6
50–59	25	23	36	84	126.04
60–69	36	33	40	109	120.49
70–79	21	5	21	47	115.77
80–89	5	1	7	13	123.85
5–99	0	1	0	1	116.40
Total	103	78	130	311	Mean 122.93

**Table 6:** TCHOL level data for Type 2 diabetes patients at the Mbeya Zonal Referral Hospital.

**Gender**	**T** **C** **H** **O** **L** < 200	**TCHOL ≥200 and < 240**		**T** **C** **H** **O** **L** ≥ 240	**Total**
Male	95	30		18	143
Female	92	46		30	168
Total	187 (60.1%)	76 (24.5%)		48 (15.4%)	311

**Age group**	**T** **C** **H** **O** **L** < 200	**T** **C** **H** **O** **L** ≥ 200** and < 240**	**T** **C** **H** **O** **L** ≥ 240	**Total**	**Mean (median) TCHOL level (mg/dL)**
30–39	7	2	2	11	198.21
40–49	26	11	9	46	195.47
50–59	49	23	12	84	192.26
60–69	69	24	16	109	188.56
70–79	29	12	6	47	180.73
80–89	6	4	3	13	194.51
90–99	1	0	0	1	183.68
Total	187 (60.1%)	76 (24.5%)	48 (15.4%)	311	Mean 190.48

## Data Availability

The data that support the findings of this study are available within the article. Additional data that were generated or analyzed during the study are available from the corresponding author upon reasonable request.
